# Molecular and clinical studies in 107 Noonan syndrome affected individuals with *PTPN11* mutations

**DOI:** 10.1186/s12881-020-0986-5

**Published:** 2020-03-12

**Authors:** Jeevana Praharsha Athota, Meenakshi Bhat, Sheela Nampoothiri, Kalpana Gowrishankar, Sanjeeva Ghanti Narayanachar, Vinuth Puttamallesh, Mohammed Oomer Farooque, Swathi Shetty

**Affiliations:** 1 Molecular Genetics, Centre for Human Genetics, Bengaluru, 560100 India; 2 Pediatric Genetics, Indira Gandhi Institute of Child Health, Bengaluru, 560029 India; 3 Pediatric Genetics, Amrita Institute of Medical Sciences & Research Centre (AIMS), Kochi, 682041 India; 4 Medical Genetics, Kanchi Kamakoti CHILDS Trust Hospital, Chennai, 600034 India

**Keywords:** Noonan syndrome, PTPN11, Mutational analysis, Congenital heart defects, SHP-2, RASopathy

## Abstract

**Background:**

Noonan syndrome (NS), an autosomal dominant developmental genetic disorder, is caused by germline mutations in genes associated with the RAS / mitogen-activated protein kinase (MAPK) pathway. In several studies *PTPN11* is one of the genes with a significant number of pathogenic variants in NS-affected patients. Therefore, clinically diagnosed NS individuals are initially tested for pathogenic variants in *PTPN11* gene to confirm the relationship before studying genotype–phenotype correlation.

**Methods:**

Individuals (363) with clinically diagnosed NS from four hospitals in South India were recruited and the exons of *PTPN11* gene were sequenced.

**Results:**

Thirty-two previously described pathogenic variants in eight different exons in *PTPN11* gene were detected in 107 patients, of whom 10 were familial cases. Exons 3, 8 and 13 had the highest number of pathogenic variants. The most commonly identified pathogenic variants in this series were in exon 8 (c.922A > G, c.923A > G), observed in 22 of the affected. Congenital cardiac anomalies were present in 84% of the mutation-positive cohort, the majority being defects in the right side of the heart. The most common facial features were downward-slanting palpebral fissures, hypertelorism and low-set posteriorly rotated ears. Other clinical features included short stature (40%), pectus excavatum (54%) and, in males, unilateral or bilateral cryptorchidism (44%).

**Conclusion:**

The clinical features and mutational spectrum observed in our cohort are similar to those reported in other large studies done worldwide. This is the largest case series of NS-affected individuals with *PTPN11* mutations described till date from India.

## Background

Worldwide a number of molecular studies have shown involvement of *PTPN11* in causation of Noonan syndrome (NS). Our cohort of 363 patients from South India is one of the largest series described. The only other study on Indian patients with NS was published in 2017 by Phadke et al. [1], who screened 17 patients for *PTPN11* and found pathogenic variants in 11 patients.

RASopathies are a well-documented group of autosomal-dominant disorders that result from mutations in genes in the RAS / mitogen-activated protein kinase (MAPK) pathway. Thirty genes have so far been implicated in the six different syndromes that are grouped as RASopathies [2, 3]. These syndromes are clinically heterogeneous, with overlapping features that complicate diagnosis without genetic testing. NS [MIM 163950] is the most common RASopathy, with a prevalence rate in livebirths of 1:1000 to 1:2500 [4]. It is postulated that a proportion of NS patients may be underdiagnosed owing to milder expression of the syndrome [5].

NS was first categorized as a separate entity in 1968 [6], with distinctive clinical features including facial dysmorphology, short stature and cardiac abnormalities [7, 8] (mainly pulmonary stenosis and patent ductus arteriosus). Numerous studies since then have shown that NS varyingly encompasses other features of skeletal, neurological, endocrine, haematological and cutaneous abnormalities [9]. Clinical features such as pulmonary stenosis (PS), short stature and thoracic deformities are more consistently associated with *PTPN11* mutations than other clinical features [10–14]. The association of congenital heart defects in some studies is greater than 80% [1, 10, 11, 13–30] indicating that NS is the second syndrome after Down syndrome with respect to frequency of cardiac defects.

Features of facial dysmorphism in NS include curly/coarse/sparse hair, hypertelorism, downward slanting palpebral fissures, ptosis, strabismus, broad flat nose, high arched palate, micrognathia, short webbed neck and low-set posteriorly rotated ears, some of which are less distinguishable with age [31, 32]. Widely spaced and/or low-set nipples, pectus excavatum, pectus carinatum, scoliosis, cryptorchidism (in males), menorrhagia (in females), and neurocutaneous markers such as lentigines and café au lait spots are some of the other associated features. One of the studies reported pragmatic language impairment [PLI] in children with NS. People with PLI have difficulties in understanding and adapting to the needs of their conversational partners [33].

NS is also detectable prenatally by the presence of cystic hygromas, pleural effusions, polyhydramnios (33%) with or without hydrops foetalis, congenital heart disease, chorioangiomas and other ultrasonographic markers [34]. A diagnosis of NS should be considered in a foetus observed to have significantly increased nuchal translucency with normal chromosomes [13, 34].

**Fig. 1:**
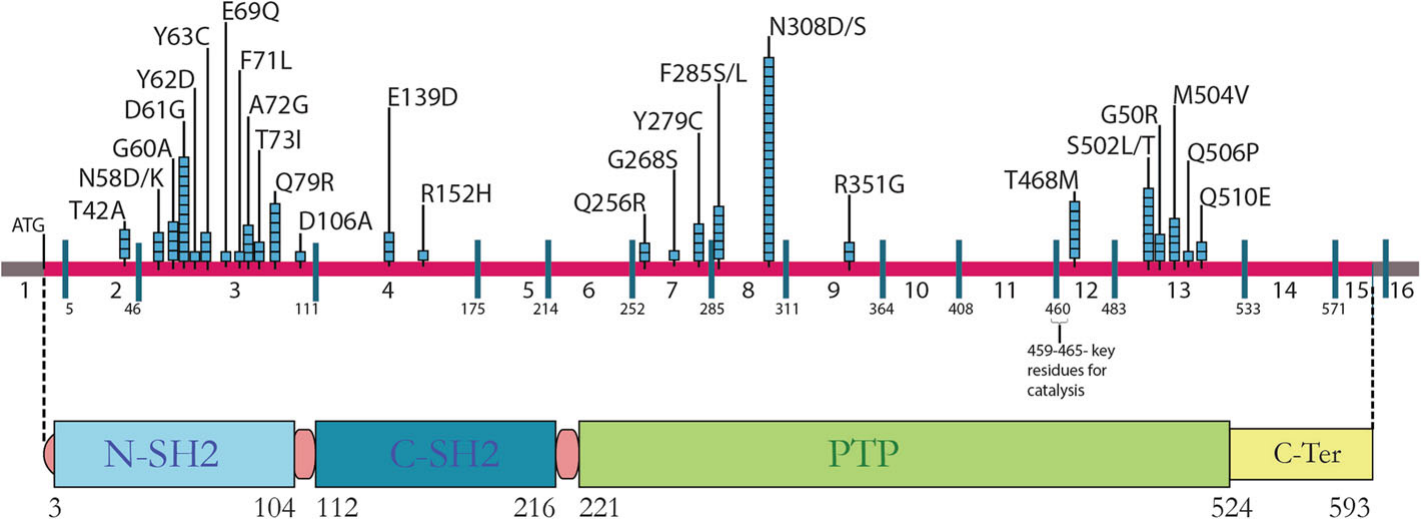
Location of *PTPN11* pathogenic variants shown along with exons and SHP-2 functional protein domains. The bar at top depicts the exons of the *PTPN11* gene, with the coding region in pink. The blue boxes above the bar represent the number of patients observed with that particular pathogenic variant. The corresponding functional domains of the SHP-2 protein are shown below and includes the two SH2 domains and the protein tyrosine phosphatase (PTP) domain

Fourteen different genes (*PTPN11, KRAS, SOS1, RAF1, NRAS, BRAF, SHOC2, CBL, RRAS, RIT1, LZTR1, SOS2, MEK1 and PPPICB*) [35–48] have thus far been implicated in aetiology of NS. Of these, the most commonly mutated gene in NS is *PTPN11*, with mutation frequency ranging between 22 and 100% [10, 20, 23] across studies. *PTPN11* codes for a ubiquitous non-receptor tyrosine phosphatase, the Src homology region 2 (SH2) domain-containing tyrosine phosphatase 2 or SHP2 protein. The structure and function of this protein, a member of the RAS/MAPK cascade, are highly conserved from invertebrates to mammals [49, 50]. In humans, *PTPN11* comprises 16 exons, with exon 1 consisting of an untranslated 5′ region and the translation initiation codon ATG, and exons 15 and 16 containing the stop codon TGA and 3′ untranslated region respectively (see Fig. 1). *PTPN11* translates into a 593 amino acid protein comprising four distinct domains, two tandem SH2 domains (N-terminal SH2 and C-terminal SH2), a central PTP domain, and a C-terminal hydrophilic tail [51]. The SH2 domains are responsible for the interaction with phosphotyrosine-containing activators which contain binding sites for the N-SH2 domain. The PTP domain includes four loops, namely the P loop, pY loop, WPD loop and Q loop, which together enclose the ‘active site pocket’ and have specific roles in SHP2 activity. The residues C459(X_5_) and R465 are important for catalysis. Stabilization of the phosphotyrosine substrate and enzyme complex is brought about by the R465 residue while C459 is responsible for a nucleophilic attack on the phosphorus atom leading to a transfer of the phosphate group from the substrate to the enzyme.

In the basic state there exists a low-level interaction between N-SH2 and the catalytic site of the PTP domain, maintaining an auto-inhibitory closed conformation. To be catalytically activated, this closed conformation has to be ‘broken’, allowing an ‘open’ configuration and entry of the substrate into the catalytic site. The open conformation is brought on by specific interactions between sites on the N-SH2 domain and upstream-signalling molecules. The conformational change thereby functions as a molecular switch turned on only upon interaction with signalling partners [49, 52]. This ability allows the protein to function as a Ras/MAPK extracellular-regulated kinases 1/2 (ERK1/2) cascade stimulator on interaction with various activating molecules or agonists [53]. The Ras/MAPK pathway is involved in cell proliferation, differentiation, metabolism, apoptosis and cell survival. The pathway is a cascade, controlled by the expression of various genes in a delicate balance, and a molecular aberration in any one of these deregulates the pathway causing multiple developmental anomalies [2, 50, 54, 55].

NS and Noonan syndrome with multiple lentigines (NSML, MIM *151100) are the result of a gain-of-function mutation in *PTPN11* [52]. Conversely, loss-of-function mutations have also been identified, which cause a skeletal disorder called metachondromatosis (MC; MIM#156250).

We report here findings of our study of 363 patients clinically diagnosed as having Noonan or Noonan-like syndromes.

## Methods

### Clinical

We used a cohort of 363 patients from four hospitals in South India identified as NS patients by clinical geneticists using the van der Burgt scoring system [31]. A detailed pro forma record including three-generation pedigree, prenatal ultrasonographic findings, anthropometry and clinical features was prepared for each family. Venous blood samples were collected for DNA testing after informed assent/consent from the affected individuals and parents. Clinical photographs were taken where families had consented.

The characteristic features used in diagnosis included curly coarse hair, broad forehead, hypertelorism, downward slanting palpebral fissures, unilateral or bilateral ptosis, and low-set posteriorly rotated ears. Other clinical features recorded were neck webbing, widely spaced nipples, pectus excavatum or pectus carinatum, cryptorchidism (in males) and neurocutaneous markers.

### Molecular studies

DNA was isolated from peripheral blood samples. Three hundred and sixty-three samples which met the required clinical criteria of NS were processed. The DNA was used to amplify exons 2–16 of the *PTPN11* gene by PCR amplification see (Additional file 1). The amplified products were purified, Sanger sequenced (Applied Biosystems ABI 3500), and analysed using Sequencher software®.

### Analysis

All the *PTPN11* variants observed have been verified as reported pathogenic variants in HGMD (Human Gene Mutation Database) and also cross-verified with the NCBI database.

## Results

**Fig. 2:**
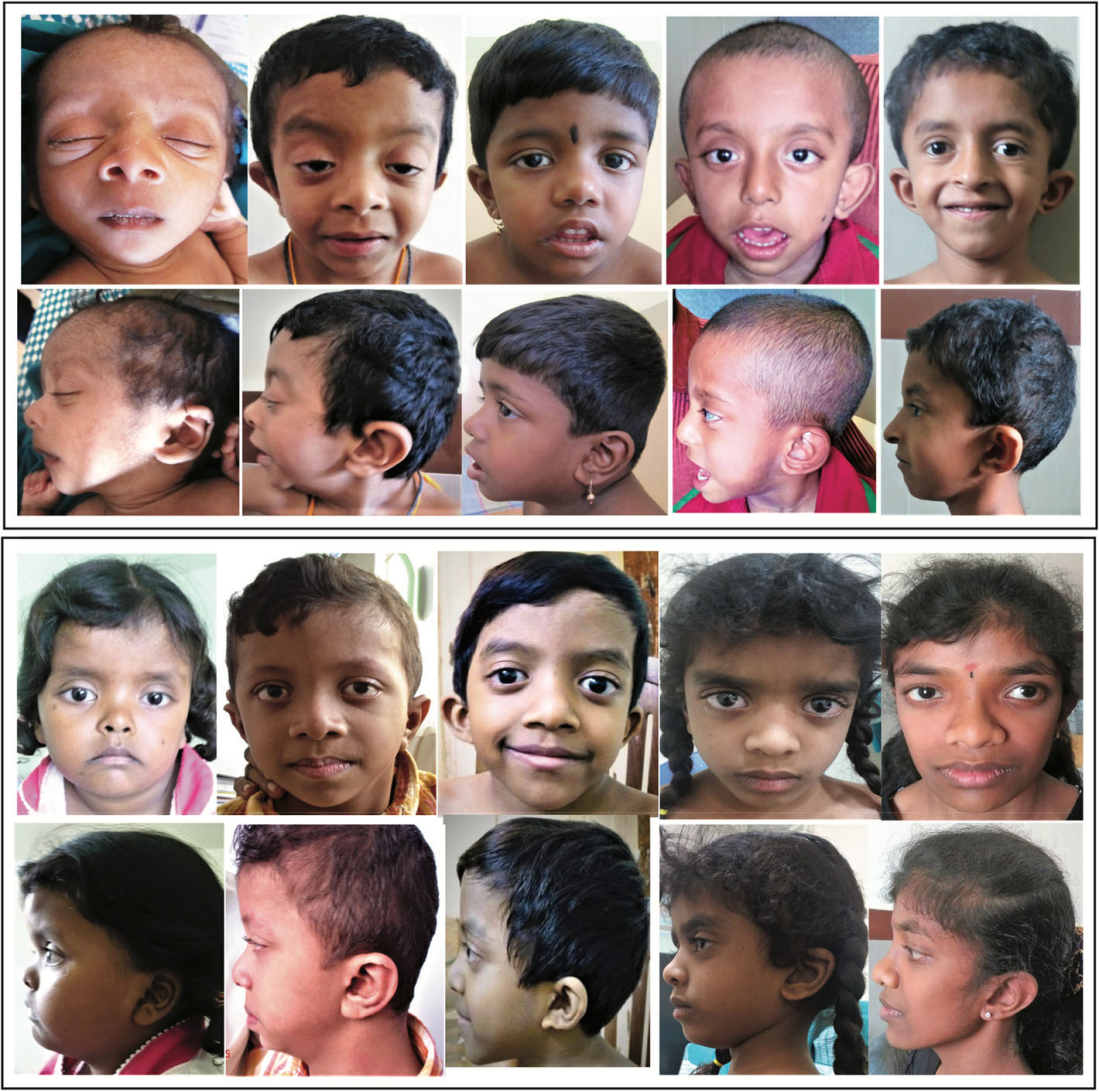
Typical facial features (face front and side) in *PTPN11* pathogenic variant positive NS patients. Curly/sparse hair, hooded eyes / ptosis, down-slanting palpebral fissures, hypertelorism and low-set, posteriorly rotated ears seen at ages ranging from 3 months to16years

Exon sequencing of *PTPN11* gene revealed that 107 of the 363 (29%) patients harboured a heterozygous pathogenic variant (Fig. 1). Clinical features of these individuals are described in Table 1. Age at diagnosis ranged between 28 days and 32 years (Fig. 2), and 61% of patients were males and 39% females. A predilection for males among patients brought for medical evaluation earlier and more frequently is the norm in most Indian situations and this may account for the sex discrepancy in our cohort.

**Table 1:** Clinical features in PTPN11 pathogenic variant positive patients in the present study

**Craniofacial characteristics**	**Percentage of*PTPN11*positive patients**	**Cardiac defects**	**Percentage of*PTPN11*positive patients**
Broad/High forehead	31.7	Hypertrophic cardiomyopathy	8.5
Hypertelorism	50	Atrial septal defect	35.3
Ptosis	54.8	Ventricular septal defect	4.8
Downward Slanting palpebral fissures	84	Pulmonary stenosis	35.3
Low set ears	67		
**Systemic Characteristics**	**Percentage of*PTPN11*positive patients**	**Skin/Hair Abnormalities**	**Percentage of*PTPN11*positive patients**
Short Stature	40	Dry Skin	4.8
Webbed Neck/Low Posterior Hairline	60.9	Curly hair/woolly hair	8.5
Pectus abnormalities	53.6	Café au lait patches	9.75
Widely spaced nipples	34.14		
Cubitus Valgus	12.19		
Cryptorchidism	43.75

In 10 families, one parent was found to be clinically affected.

**Fig. 3:**
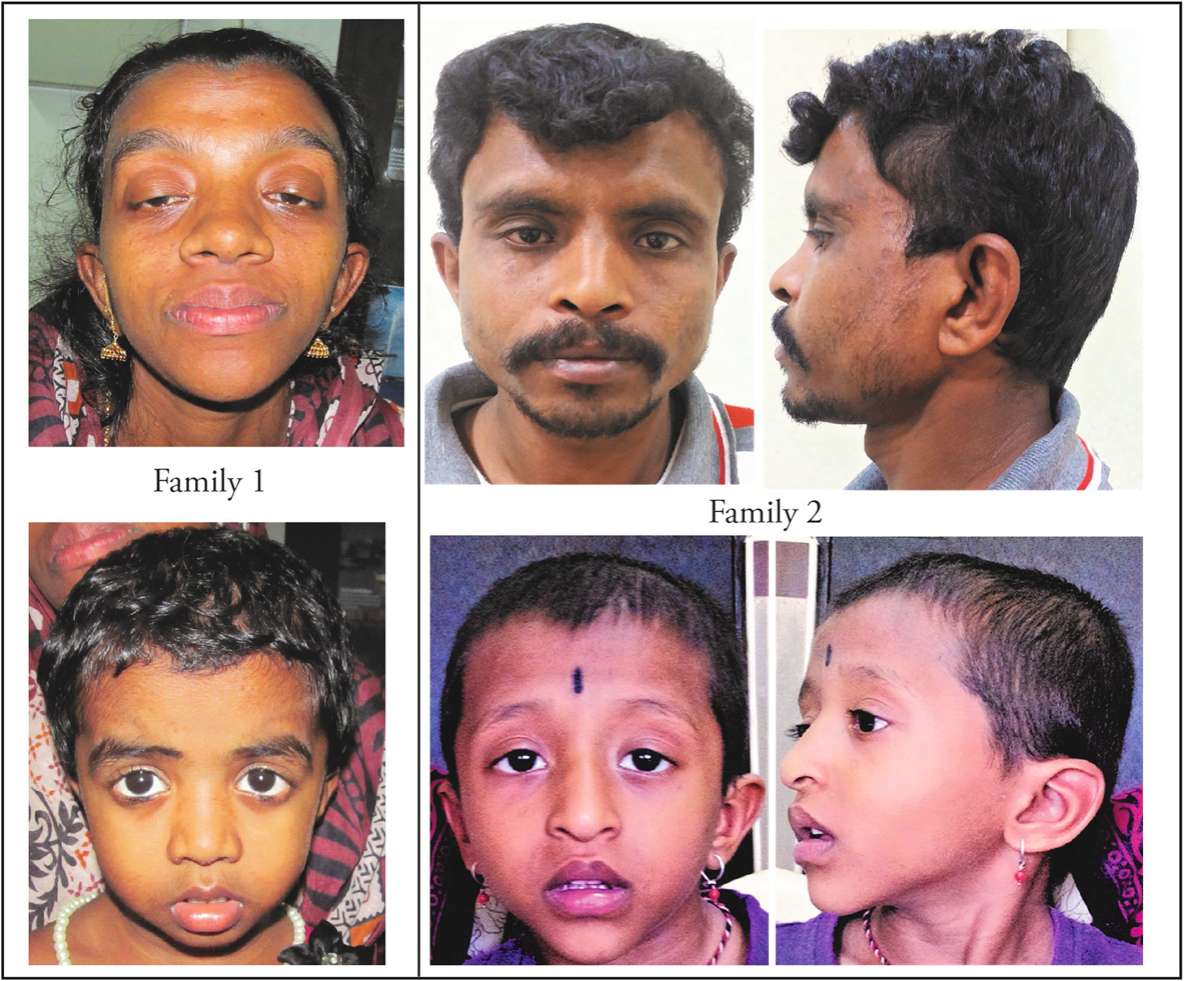
Facial features observed in two NS families. Family 1-Mother and daughter with the pathogenic variant c.923A > G in exon 8 of the *PTPN11* gene. Family 2- Father and son with the c.922A > G pathogenic variant in exon 8 of the *PTPN11* gene

We identified a total of 32 different *PTPN11* variants in 107 patients (Table 2), all of which have been reported earlier as pathogenic. As in earlier studies, exons 3 and 8 harboured the highest number of pathogenic variants (Fig. 1). The change c.922A > G (p.Asn308Asp) was the most common pathogenic variant observed in our patient set (11.21%); the frequency of protein variants at residue 308 increases to 21% if the change p.Asn308Ser, which results from a mutation in the same codon (c.923A > G), is included (Table 2). This is consistent with findings from other studies [11, 24, 30]. Eighteen (18) parent sets of the positive cases were screened for the pathogenic variant found in the proband and in 10 families one parent was positive for the pathogenic variant that was seen in the proband (Fig. 3).

**Table 2:** *PTPN11* pathogenic variants^a^ and their numbers observed in our study

Exon number	Positive sample number	Variation seen	Number of times observed	Amino acid change
Exon 2	3	c.124 A > G	3	p.Thr42Ala
Exon 3	38	c.172A > G	3	p.Asn58Asp
		c.174 C > G	1	p.Asn58Lys
		c.179 G > C	4	p.Gly60Ala
		c.181 G > A	4	p.Asp61Asn
		c.188 A > G	3	p.Try63Cys
		c.182 A > G	7	p.Asp61Gly
		c.184 T > G	1	p.Tyr62Asp
		c.205 G > C	1	p.Glu69Gln
		c.211 T > C	1	p.Phe71Lue
		c.214 G > T	2	p.Ala72Ser
		c.215 C > G	2	p.Ala72Gly
		c.218 C > T	2	p.Thr73Ile
		c.236 A > G	6	p.Gln79Arg
		c.317 A > C	1	p.Asp106Ala
Exon4	4	c.417 G > C	3	p.Glu139Asp
		c.455G > A	1	p.Arg152His
Exon7	7	c. 767 A > G	2	p.Gln256Arg
		c.802 G > A	1	p.Gly268Ser
		c.836 A > G	4	p.Tyr279Cys
Exon8	28	c.854 T > C	4	p.Phe285Ser
		**c.922A > G**^**b**^	**12**	**p.Asn308Asp**
		**c.923 A > G**^**b**^	**10**	**p.Asn308Ser**
		c.855 T > G	2	p.Phe285Leu
Exon 9	2	c.1052 G > A	2	p.Arg351Gln
Exon 12	6	c.1403C > T	6	p.Thr468Met
Exon 13	19	c.1504 T > A	5	p.Ser502Thr
		c.1505 C > T	3	p.Ser502Leu
		c.1507 G > A	3	p.Gly503Arg
		c. 1510 A > G	5	p.Met504Val
		c.1517 A > C	1	p.Gln506Pro
		c.1528 C > G	2	p.Gln510Glu

^a^In the current study 32 different *PTPN11 pathogenic* variations were observed

^b^The 2 most common variations observed affecting the same amino acid residue

A large phenotypic variability is observed among NS patients with the same pathogenic variant, indicating variable expressivity and penetrance [56, 57]. Sixty–seven of the patients with pathogenic variants in *PTPN11* had congenital heart defects (84%), with pulmonary stenosis and secundum type atrial septal defect each accounting for 35.3% of the cardiac anomalies. Hypertrophic cardiomyopathy (HCM) was seen in 8.5% and ventricular septum defect (VSD) in 4.8%of the patients. Congenital heart defects (CHD) were seen most frequently in patients with pathogenic variants in exon 3 (28 out of 38 patients) and exon 8 (18out of 28 patients) of the *PTPN11* gene see (Additional file 2). 21(19.06%) patients out of 107 patients with *PTPN11* pathogenic variants did not have cardiac issues or had not undergone any cardiac evaluations.

The other features observed with high frequency in the patients with *PTPN11* pathogenic variants were downward slanting palpebral fissures (84%), low-set posteriorly rotated ears (67%) and webbed neck (61%). Sternal deformities (pectus excavatum and pectus carinatum) were seen in 54% and in combination with pulmonary stenosis was observed in 30% of the pathogenic variant-positive patients. 44% of male patients with *PTPN11* variants were observed to have unilateral or bilateral cryptorchidism. All patients with *PTPN11* pathogenic variants had near-normal developmental milestones and cognitive abilities and the majority attended mainstream school. Short stature (height < 3rd centile for age) was noted in only 40% of mutation positive patients.

## Discussion

### *PTPN11* pathogenic variants

There are now numerous studies that have screened for and reported *PTPN11* pathogenic variants in NS patients. Mutation detection rates have varied from 22 to 100% (Table 3). The table shows that there have been other studies that found a low percentage of patients with *PTPN11* pathogenic variants like the present study (29%) [15, 16, 19, 21–23, 27, 28]. This substantiates the fact that NS is a genetically heterogeneous syndrome, and may imply higher frequency of pathogenic variants among the other 13 genes known to cause NS and possibly in other, yet unidentified genes. The large-cohort study published by Ezquieta et al [27] in 2012 had 643 patients of whom 172 (27%) had pathogenic variants in *PTPN11.* Zenker et al. [11] recruited patients from the paediatric cardiology department and showed a 60% detection of pathogenic variants, suggesting that the method of patient recruitment may change pathogenic variant detection rates.

**Table 3:** Comparison of *PTPN11* pathogenic variant data and their association with heart defects in our study and in other NS studies

Study	No. of patients tested	No. of *PTPN11* positive patients (%)	Percentage of *PTPN11* positive patients with PS	Percentage of *PTPN11* Positive patients with (Septal defects) ASD + VSD	Percentage of *PTPN11* positive patients with HCM
Atik et al. 2015 [10]	20	20 (100)	55	5	10
Bertola et al. 2006 [26]	61	26 (42.6)	67	–	10
Cizmarova et al. 2016 [14]	51	22 (43)	63	31.79	9.09
Dallapicola et al. 2003 [13]	84	34(40)	41	5.90	23.05
Ezquieta et al. 2012 [27]	643	172(27)	49	–	3.48
Ferrero et al 2008 [28]	40	14 (31.5)	85.70	7.14	7.14
Hung et al. 2007 [29]	34	13(38)	–	–	–
Jongmans et al. 2005 [30]	170	76(45)	68	30	7
Kiper et al. 2012 [15]	31	9(28)	55	33	11.10
Kosaki et al. 2002 [16]	21	7 (33)	42	42	–
Louati et al 2014 [17]	19	9 (43)	88	22.20	–
Maheshwari et al. 2002 [18]	16	8(50)	75	18.75	–
Min Ko et al. 2008 [19]	59	16(27)	50	36	18
Mona L. Essawi et al. 2013 [20]	21	21(100)	24	–	19
Musante et al. 2002 [21]	96	32(33)	70	23	–
Papadopoulous et al. 2011 [22]	60	17(29)	65	17	–
Rodriguez et al 2014 [23]	18	4(22)	25	–	–
Shuba Phadke et al. 2017 [1]	17	11(64.7)	54	36.36	–
Tartaglia et al. 2002 [24]	119	54(45)	70	12	5.90
Yoshida et al. 2004 [25]	45	18(40)	88	88	–
Zenker et.al 2004 [11]	57	34(60)	88	94	26.40
Current study	**363**	**107(29)**	**35.3**	**40.01**	**8.5**

*PS* Pulmonary stenosis, ASD – Atrial septal defect; VSD – Ventricular septal defect; HCM – Hypertrophic cardiomyopathy

All the pathogenic variants identified in our cohort are previously reported missense changes and occur at positions that are conserved among vertebrate *PTPN11* orthologous genes. A majority of these are in exons 3 and 8, as reported in earlier studies, and account for 62% of the *PTPN11* pathogenic variants (Fig. 1). In the present study 91 of the 107 patients (85%) with pathogenic variants have the variants in exons 3, 8, 12 and 13; this proportion is consistent over most studies [11, 13, 19, 21, 30]. The change c.922A > G, which results in p.Asn308Asp, was the most common pathogenic variant we observed. This is consistent with most of the other *PTPN11* mutation studies and therefore position 922 in *PTPN11* can be regarded as a mutation hotspot. The amino acid residue 308 lies in the PTP domain of the protein (which contains four loops, P loop, pY loop, WPD loop and Q loop) and is not involved in direct interactions with the N-SH2 domain [58]. The Asp at this position forms extra hydrogen bonds with pY loop residues making the pY and the P loops less flexible and renders the enzyme better configured for catalysis by maintaining the open conformation. The extent of rigidity or flexibility between the pY and P loops determines the ability of the N-SH2 domain to close back upon the PTP domain once it is in the open configuration. Keilhack et al [59] showed that the Asn308Asp change caused the enzyme to have increased basal as well as stimulated activity compared to the wild-type enzyme. This feature of the SHP2 protein, where the open or unlocked state allows the catalytic function, explains the gain-of-function mechanism of most of the mutations seen in *PTPN11* that cause locking up of the enzyme in the open state. This is due to various interactions that are gained or lost owing to the amino acid change. In another mechanism for locking the enzyme in the open configuration, the Asp61Gly mutation in the N-SH2 domain weakens its interaction with the PTP domain, leaving the enzyme in the active catalytic state [60].

The absence of frameshift, nonsense or even splicing mutations in our cohort and in other similar studies implies that there is no haploinsufficiency in *PTPN11* expression and that *PTPN11* haploinsufficiency does not cause NS. Lee et al [61] and Yoshida et al [25] reported frameshift mutations that cause the changes Asp61del and Gly60del and showed that the changes resulted in an opening of the catalytic site, therefore functioning via positive upregulation as opposed to haploinsufficiency of the wild-type gene. Three splicing mutations reported earlier by Bowen et al [62] are associated with metachondrochromatosis, which is a result of loss-of-function mutations. This study identified five frameshift, two nonsense, three splice-site, and one large-deletion mutations in 11 families, with each family harbouring a different mutation and the mutations identified were spread throughout the gene.

### Genotype–phenotype correlation

Nearly all studies carried out to date on NS mutation testing have attempted genotype–phenotype correlation. However, the most consistent feature across studies has been that there exists a great deal of phenotypic variation even among patients with the same pathogenic variant [56]. Several *PTPN11* functional studies have addressed different developmental aspects and shed some light on the probable causes of such variation based on SHP2 functionality [49, 52, 63].

Among mutation-positive patients 40% exhibited short stature (height < 3rd centile for age). This may be because the majority of patients were young children seen before pubertal growth had been completed [64].

Characterization of facial features can vary as this is still based on subjective assessment [17]. The most commonly observed facial features in our study were downward slanting palpebral fissures followed by low-set posteriorly rotated ears. A recent study by Kruszka et al (2017) [65] used facial analysis technology to assess facial deformities associated with NS. This may prove to be the way forward to overcome subjective assessment bias.

Eight patients in our cohort had café au lait spots / multiple lentigines; three of them had pathogenic variants in exon 3 (c.182A > G, c.188A > G and c.236A > G), two patients in exon 4 (c.417G > C), two in exon 7 (c.767A > G and c.836A > G), and one patient with a pathogenic variant in exon 13 (c.1504 T > A). Earlier reports of NS patients with café au lait spots / multiple lentigines did not report pathogenic variants in exon 3 or exon 4. Of the six patients with c.1403C > T (p.Thr468Met) known to be associated with Noonan like syndrome with multiple lentigens, only one showed presence of multiple lentigines but three others had thick curly or woolly hair. As lentigines increase with age and the majority of our patients were younger than 5 years, the frequency of patients with lentigines in our study may be lower than that in other studies. Thirteen patients with mutations in exons 7, 12 and 13, some of which are associated with LEOPARD syndrome were identified. Of these, only two patients (with mutations in exons 7 and 12) had the classic features, including deafness, cardiac rhythm abnormalities and widespread multiple lentigines. There was also a high correlation of patients with pectus deformity with pathogenic variants in exon 13 (10 out of 19 patients) see (Additional file 2).

Our cohort had CHD (in 84% of patients with pathogenic variants) mainly consisting of right-sided cardiac anomalies [57]. Studies by Zenker [11], Tartaglia [66], Louati [17] and Ferrero [28] also reported high frequency of CHD. The most frequently occurring pathogenic variants in our cohort, c.922A > G and c.923A > G, both of which change the same amino acid (Asn308), were seen in 22 patients, including a three-generation-affected pedigree.

We did not detect any pathogenic variants in the C terminal region, which is consistent with most other studies [17].

Of the 18 sets of parents screened in our study for the causal pathogenic variant, seven fathers and three mothers carried the variant detected in the child. Three of these children had cardiac defects (two PS and one ASD) that were not present in the parents. This may be explained by intrafamilial variability with milder phenotypes in reproductively fit parents or may be as a result of cardiac involvement in parents regressing or improving with age.

## Conclusion

Although next-generation sequencing (NGS) using a RASopathy gene panel is the way forward for NS and other RASopathy testing, the cost involved might be an inhibiting factor for patients from low economic backgrounds and countries where such testing is expensive and/or not routinely available. In such settings we propose a preliminary protocol based on our findings and other similar studies. This involves sequencing of *PTPN11* exons 3 and 8 followed by 13, especially if the clinical features include heart defects, downward slanting palpebral fissures, webbed neck, low-set posteriorly rotated ears and pectus abnormalities.

No single clinical feature was seen consistently across all patients, confirming the wide variability observed among NS patients. Most of the clinical findings reported in our cohort are in keeping with those in other large studies reported in the literature [67–69].

To our knowledge, this report is the largest series of *PTPN11* variant-positive cases reported in Asian Indian individuals. The 256 patients who were found to be *PTPN11* variant-negative are the subject of an NGS panel study involving 22 other genes implicated in causation of NS and other RASopathies.

## Supplementary information

**Additional file 1.** Primers for exon sequencing.

**Additional file 2.** Exon-wise comparison of the clinical features.

## Data Availability

All sanger sequencing data generated and analysed in the current study are available on the Genbank database. Accession numbers are MT052193-MT052299.

## Abbreviations

CHD: Congenital heart defects
NS: Noonan syndrome
NT: Nuchal translucency
PLI: Pragmatic language impairment
PTPN11: Protein tyrosine phosphate non-receptor type11
