# Supplementary material for: Molecular and clinical studies in 107 Noonan syndrome affected individuals with PTPN11 mutations
Source: BMC Med Genet. 2020 Mar 12;21:50. doi: 10.1186/s12881-020-0986-5 (PMC7068896; doi:10.1186/s12881-020-0986-5)
Supplement: Supplementary file 1 — Additional file 1. Primers for exon sequencing. [file 12881_2020_986_MOESM1_ESM.pdf]

Additional File 2: Primers for Exon sequencing [24]

PRIMER NAME	PRIMER SEQUENCES
PTPN11 exon 1F	GCTGACGGGAAGCAGGAAGTGG
PTPN11 exon 1R	CTGGCACCCGTGGTTCCCTC
PTPN11 exon 2F	ACTGAATCCCAGGTCTCTACCAAG
PTPN11 exon 2R	CAGCAAGCTATCCAAGCATGGT
PTPN11 exon 3F	CGACGTGGAAGATGAGATCTGA
PTPN11 exon 3R	CAGTCACAAGCCTTTGGAGTCAG
PTPN11 exon 4F	TTGATCAATCCCTTGGAGGA
PTPN11 exon 4R	CATCTGTAGGTGATAGAGCAAGA
PTPN11 exon 5F	CTGCAGTGAACATGAGAGTGCTTG
PTPN11 exon 5R	GTTGAAGCTGCAATGGGTACATG
PTPN11 exon 6F	TGCATTAACACCGTTTTCTGT
PTPN11 exon 6R	GTCAGTTTCAAGTCTCTCAGGTC
PTPN11 exon 7F	GAACATTTCCTAGGATGAATTCC
PTPN11 exon 7R	GGTACAGAGGTGCTAGGAATCA
PTPN11 exon 8F	GACATCAGGCAGTGTTCACGTTAC
PTPN11 exon 8R	CCTTAAAGTTACTTTCAGGACATG
PTPN11 exon 9F	GTAAGCTTTGCTTTTCACAGTG
PTPN11 exon 9R	CTAAACATGGCCAATCTGACATGTC
PTPN11 exon 10F	GCAAGACTTGAACATTTGTTTGTTGC
PTPN11 exon 10R	GACCCTGAATTCCTACACACCATC
PTPN11 exon 11F	CAAAAGGAGACGAGTTCTGGGAAC
PTPN11 exon 11R	GCAGTTGCTCTATGCCTCAAACAG
PTPN11 exon 12F	GCTCCAAAGAGTAGACATTGTTTC
PTPN11 exon 12R	GACTGTTTTCGTGAGCACTTTC
PTPN11 exon 13F	CAACACTGTAGCCATTGCAACA
PTPN11 exon 13R	CGTATCCAAGAGGCCTAGCAAG
PTPN11 exon 14F	ACCATTGTCCCTCACATGTGC
PTPN11 exon 14R	CAGTGAAAGGCATGTGCTACAAAC
PTPN11 exon 15F	CAGGTCCTAGGCACAGGAACTG
PTPN11 exon 15R	ACATTCCCAAATTGCTTGCCT
