# Supplementary material for: Molecular and clinical studies in 107 Noonan syndrome affected individuals with PTPN11 mutations
Source: BMC Med Genet. 2020 Mar 12;21:50. doi: 10.1186/s12881-020-0986-5 (PMC7068896; doi:10.1186/s12881-020-0986-5)
Supplement: Supplementary file 2 — Additional file 2. Exon-wise comparison of the clinical features. [file 12881_2020_986_MOESM2_ESM.pdf]

Additional File 1: Exon-wise comparison of the clinical features

Exon Number	Broad forehead	Hypertelorism	Ptosis	Downward slanting palpebral fissures	Low set ears	Webbed neck	ASD	VSD	PS	HCM	Short stature	Cubitus Valgus	Pectus	Widely spaced nipples	Café au lait spots
**Exon 2**	2/3	2/3	3/3	3/3	3/3	2/3	1/3		2/3		3/3	1/3	1/3		
**Exon3**	5/38	15/38	13/38	23/38	16/38	18/38	12/38	1/38	14/38	1/38	10/38				3/38
**Exon 4**	1/4	2/4	2/4	3/4	3/4	1/4	1/4	1/4	2/4		1/4	1/4	2/4	1/4	
**Exon7**	3/7	3/7	3/7	5/7	3/7	4/7	2/7			3/7	1/7		3/7	2/7	2/7
**Exon 8**	6/28	10/28	11/28	17/28	15/28	12/28	7/28		9/28	2/28	10/28	4/28	8/28	4/28	
**Exon9**	1/2	1/2			1/2	1/2					1/2				
**Exon 12**	3/6	1/6	3/6	6/6	4/6	4/6	1/6		1/6	1/6	2/6	1/6	3/6	2/6	
**Exon 13**	5/19	7/19	10/19	12/19	10/19	10/19	5/19		4/19	2/19	5/19	2/19	10/19	5/19	1/19
**# 922 A>G Exon 8**	3/12	7/12	4/12	12/12	10/12	7/12	4/12		5/12	2/12	5/12	3/12	5/12	4/12	

The variation that is most commonly seen in *PTPN11* positive samples; PS – Pulmonary stenosis; ASD – Atrial septal defect; VSD – Ventricular septal defect; HCM – Hypertrophic cardiomyopathy
